# Enhanced sensitivity to androgen withdrawal due to overexpression of interleukin-6 in androgen-dependent human prostate cancer LNCaP cells

**DOI:** 10.1038/sj.bjc.6605358

**Published:** 2009-10-20

**Authors:** T Terakawa, H Miyake, J Furukawa, S L Ettinger, M E Gleave, M Fujisawa

**Affiliations:** 1 Division of Urology, Department of Surgery, Kobe University Graduate School of Medicine, Kobe, Japan; 2 The Prostate Centre, Department of Surgery, Vancouver General Hospital, Vancouver, Canada

**Keywords:** prostate cancer, interleukin-6, androgen-independent progression, androgen withdrawal, signal transduction

## Abstract

**Background:**

The objective of this study was to investigate the effects of interleukin-6 (IL-6) overexpression in androgen-dependent prostate cancer LNCaP cells on their phenotype under an androgen-deprived condition.

**Methods:**

We established IL-6-overexpressing LNCaP (LNCaP/IL-6) by introducing the expression vector containing IL-6 cDNA. Changes in the phenotype in LNCaP/IL-6 were compared with that in LNCaP transfected with control vector alone (LNCaP/Co).

**Results:**

*In vitro*, the growth of LNCaP/IL-6 was significantly inferior to that of LNCaP/Co under an androgen-deprived condition. Similarly, LNCaP/IL-6 tumour in nude mice rapidly regressed after castration; however, LNCaP/Co tumour growth was transiently inhibited after castration and then continuously accelerated. After androgen withdrawal, expression levels of phosphorylated p44/42 mitogen-activated protein kinase (MAPK) and Akt in LNCaP/IL-6 were markedly upregulated compared with those in LNCaP/Co; however, additional treatment with specific inhibitor of the MAPK or Akt signalling pathway significantly inhibited the growth of LNCaP/IL-6 compared with that of LNCaP/Co. Furthermore, gene microarray analyses showed that androgen deprivation resulted in differential expression of genes involved in growth, apoptotsis and tumorigenesis between LNCaP/Co and LNCaP/IL-6.

**Conclusion:**

Excessive secretion of IL-6 by LNCaP cells in an autocrine manner may have a suppressive function in their growth and acquisition of androgen-independent phenotype under an androgen-deprived condition.

The prognosis of patients with organ-confined prostate cancer has been remarkably improved with technical advances in surgical as well as radiological treatments; however, androgen withdrawal therapy remains the only effective form of systemic therapy for men with advanced prostate cancer. Initially, >80% of such patients respond favourably to this therapy; however, progression to the lethal and untreatable stage of androgen independence ultimately occurs within a few years in the majority of these patients (Rosenberg and Small, 2003). Recently completed phase III trials first demonstrated a survival benefit of docetaxel-based combined regimen in patients with hormone-refractory disease, but these improved effects were not substantial (Petrylak *et al*, 2004; Tannock *et al*, 2004). Collectively, these findings suggest that androgen-independent (AI) progression remains the major obstacle to effective control and cure of advanced disease, emphasising the need for a novel therapeutic strategy that targets the molecular mechanism mediating emergence of the AI phenotype.

Despite intensive efforts in the field of prostate cancer research, the precise molecular mechanism of progression to androgen independence has not been fully elucidated; however, several hypotheses explaining the complex process during AI progression have been reported (Arnold and Isaacs, 2002; So *et al*, 2005; Kasper and Cookson, 2006). One potentially important mechanism is the maintenance of androgen receptor (AR) signalling in hormone-refractory prostate cancer by crosstalk between AR and nonsteroidal molecules, including cytokines and growth factors (Culig *et al*, 1994; Craft *et al*, 1999; Culig, 2004; Wang *et al*, 2008). Of these, interleukin-6 (IL-6), a pleiotropic cytokine involved in the regulation of hematopoiesis, immune response, inflammation, bone metabolism and neural development (Kishimoto *et al*, 1992), has been regarded as one of the most important mediators during AI progression of prostate cancer through the ligand-independent activation of AR (Okamoto *et al*, 1997; Hobisch *et al*, 1998; Lou *et al*, 2000; Lin *et al*, 2001; Ueda *et al*, 2002a, 2002b; Lee *et al*, 2003).

The biological activities of IL-6 are mediated by the IL-6 receptor; that is, the binding of IL-6 to its receptor results in the activation of intracellular signalling, including mitogen-activated protein kinase (MAPK), phosphoinositol 3′-kinase (PI3K)/Akt and Janus-activated kinase (JAK)/signal transducers and activation of transcription (STAT) pathways (Kishimoto *et al*, 1992; Corcoran and Costello, 2003). In recent studies, it was also demonstrated that modulations of three major downstream signalling pathways, MAPK, Akt and STAT3, are involved in AI transactivation of AR by IL-6 (Yang *et al*, 2003). However, the reported actions of IL-6 in conferring malignant phenotype to prostate cancer cells remain controversial, with some studies showing positive findings (Okamoto *et al*, 1997; Hobisch *et al*, 1998; Lou *et al*, 2000; Lin *et al*, 2001; Ueda *et al*, 2002a, 2002b; Lee *et al*, 2003), and others showing negative findings (Mori *et al*, 1999; Deeble *et al*, 2001; Hobisch *et al*, 2001; Jia *et al*, 2004; Lee *et al*, 2007). It also remains controversial which signal transduction pathway is most dominantly associated with the transactivation of AR induced by IL-6 (Chen *et al*, 2000; Chung *et al*, 2000; Corcoran and Costello, 2003; Yang *et al*, 2003). For example, Chen *et al* (2000) reported that inhibition of STAT3 rather than MAPK results in inhibition of AR-mediated gene activation in response to IL-6, whereas Yang *et al* (2003) showed that IL-6 can enhance AR transactivation through both the STAT3 and MAPK pathways, but not the PI3K-Akt pathway. Furthermore, few studies have investigated the crosstalk between AR and IL-6 secreted in an autocrine manner in prostate cancer cells (Lee *et al*, 2003, 2004).

In this study, therefore, we evaluated the effects of IL-6 overexpression in human androgen-dependent (AD) prostate cancer LNCaP cells on changes in their phenotype before and after androgen withdrawal to assess the functional role of IL-6 secreted in an autocrine manner as a ligand-independent activator of AR.

## Materials and methods

### Tumour cell lines

LNCaP and PC3, derived from human prostate cancer, were purchased from the American Type Culture Collection (Rockville, MD, USA). Cells were maintained in RPMI (Life Technologies, Gaithersburg, MD, USA) supplemented with 10% heated inactivated fetal bovine serum. To investigate the androgen deprivation effect, steroid hormone-depleted charcoal-stripped medium (CSM) containing 10% CS serum was prepared as described earlier (Saeed *et al*, 1997).

### Expression plasmid and transfection to tumour cells

The cDNA fragment encoding human IL-6 was directly cloned into the expression vector pcDNA3.1 (Invitrogen, Carlsbad, CA, USA) according to the manufacturer's instructions. The pcDNA3.1/IL-6 construction was transfected into LNCaP cells by the liposome-mediated gene transfer method (Miyake *et al*, 2000a). Briefly, 2 × 10^5^ LNCaP cells were plated in a 6-cm dish, 1 day before transfection. A measure of 5 *μ*g of purified pcDNA3.1/IL-6 or pcDNA3.1 (as a control) was added to LNCaP cells after preincubation for 30 min with 5 *μ*g of lipofectamine reagent and 3 ml of serum-free OPTI-MEM (Life Technologies). Drug selection in 300 *μ*g ml^−1^ Geneticin (Life Technologies) was begun 3 days after transfection. Three weeks after the drug selection, colonies were harvested with cloning cylinders and expanded to cell lines.

### Enzyme-linked immunosorbent assay

The concentrations of IL-6 in overnight culture supernatants of 5 × 10^6^ LNCaP sublines were measured using a quantitative sandwich enzyme-linked immunosorbent assay (ELISA) kit for human IL-6 according to the manufacturer's instructions (R&D Systems, Minneapolis, MN, USA). Similarly, a sandwich ELISA kit for human prostate-specific antigen (PSA) (Zymed Laboratories, South San Francisco, CA, USA) was used to determine the concentrations of PSA in overnight culture supernatants of 5 × 10^6^ LNCaP sublines and those in sera from nude mice bearing the subcutaneous tumours of LNCaP sublines.

### Cell proliferation assay

To compare the *in vitro* proliferation of LNCaP sublines, 5 × 10^3^ cells of each cell line were seeded in each well of 12-well plates, and the number of cells in each cell line was counted daily by triplicate. In addition, the effect of androgen deprivation on the proliferation of LNCaP sublines with and without additional treatment with IL-6 (Sigma-Aldrich, Tokyo, Japan), UO126 (SA Bioscience, Frederick, MD, USA) or LY294002 (SA Bioscience) was also examined; that is, following the culture of LNCaP sublines in the standard medium for 3 days, the medium was replaced with CSM with and without supplementary IL-6, UO126 or LY294002, and the number of cells was counted.

### Western blot analysis

Western analysis was performed as described earlier (Miyake *et al*, 2000a). Briefly, samples containing equal amounts of protein (25 *μ*g) from lysates of the LNCaP sublines cultured in either standard medium or CSM were electrophoresed on an SDS–polyacrylamide gel and transferred to a nitrocellulose filter. The filters were blocked in PBS containing 5% nonfat milk powder at 4°C overnight and then incubated for 1 h with antibodies against IL-6 receptor (R&D Systems), AR, *β*-actin (Santa Cruz Biotechnology, Santa Cruz, CA, USA) and total and phosphorylated STAT3, p44/42 MAPK and Akt (Cell Signaling Technology, Danvers, MA, USA). The filters were then incubated for 30 min with horseradish peroxidase-conjugated secondary antibodies (Amersham Pharmacia Biotech, Arlington Heights, IL, USA), and specific proteins were detected using an enhanced chemiluminescence western blotting analysis system (Amersham Pharmacia Biotech).

### Gene microarray

Gene microarrays of 34 580 (70-mer) human oligos representing 24 650 genes and 37 123 gene transcripts printed on aminosilane-coated microarray slides were supplied by the microarray facility of the Prostate Centre at Vancouver General Hospital (Vancouver, Canada), and microarray analysis was performed as described earlier (Snoek *et al*, 2009). Briefly, microarrays were hybridized with 10 *μ*g of total RNA from independently prepared triplicate samples of LNCaP sublines before and after androgen deprivation, labelled with CY5 against 10 *μ*g of Universal Human Reference RNA labelled with CY3 (Stratagene, La Jolla, CA, USA), and assessed using the 3DNA Array 350 Expression Array Detection kit according to the manufacturer's instructions (Genisphere, Hatfield, PA, USA). After overnight hybridization and washing, arrays were scanned on a Scan Array Express Microarray Scanner (Perkin-Elmer, Waltham, MA, USA), and signal quality and quantity were determined using the ImaGene 8.0 software (BioDIscovery, El Segundo, CA, USA). Comparative analysis of the gene expression profiles in each cell line was conducted to identify genes that are differentially regulated by overexpression of IL-6. Genes were considered to be significantly different if the mean expression level in the IL-6-overexpressing cells was at least 2.0-fold greater or 2.0-fold less than that seen in the control cells.

### Assessment of *in vivo* tumour growth

Male athymic nude mice (BALB/c-nu/nu males, 6–8 weeks old) were purchased from Clea Japan (Tokyo, Japan) and housed in a controlled environment at 22°C on a 12-h light, 12-h dark cycle. Animals were maintained in accordance with the National Institutes of Health Guide for the Care and Use of Laboratory Animals. Each experimental group consisted of 20 mice. The tumour cells of each cell line were trypsinized, washed twice with PBS, and 5 × 10^6^ cells were subcutaneously injected with 100 *μ*l of Matrigel (Becton Dickinson, Franklin Lakes, NJ, USA) into right flank of mice. The subcutaneous tumour growth was measured once per week using calipers as described earlier (Miyake *et al*, 1996); that is, the longest surface length (a) and width perpendicular to this length (b) were measured, and tumour size was reported as a × b. Furthermore, when the tumour size was >80 mm^2^, 10 of the 20 mice were castrated, and the growth of subcutaneous tumour was continuously measured to estimate the effect of androgen withdrawal on *in vivo* growth of the LNCaP subline.

### Statistical analysis

Differences between the two groups were compared using the unpaired *t*-test. All statistical calculations were performed using Statview 5.0 software (Abacus Concepts, Inc., Berkley, CA, USA), and *P*-values <0.05 were considered significant.

## Results

### IL-6 production in LNCaP sublines

**Figure 1:**
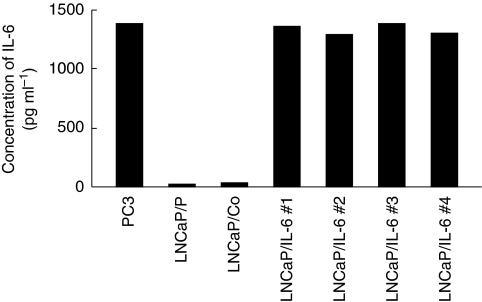
Immunoreactive interleukin-6 (IL-6) in culture supernatants of PC3 and LNCaP sublines (LNCaP/P, parental cell line of LNCaP; LNCaP/Co, control vector-only transfected cell line; LNCaP/IL-6#1, LNCaP/IL-6#2, LNCaP/IL-6#3 and LNCaP/IL-6#4, IL-6-transfected cell lines). Enzyme-linked immunosorbent assay was performed to analyse the concentrations of IL-6 protein in culture supernatant from each cell line.

LNCaP was transfected with pcDNA3.1/IL-6 or pcDNA3.1 alone as a control. After drug selection, several Geneticin-resistant stable transfectants were randomly isolated. The culture supernatants of LNCaP sublines and PC3 were analysed by sandwich ELISA to measure the IL-6 concentrations. All of the IL-6-transfected LNCaP (LNCaP/IL-6#1 to LNCaP/IL-6#4) secreted abundant immunoreactive IL-6 protein in amounts equal to those of PC3 originally expressing the IL-6 gene (Okamoto *et al*, 1997); however, limited IL-6 was detected in the supernatants of the parental LNCaP (LNCaP/P) and the cell line transfected with control vector-only transfected cell line (LNCaP/Co) (Figure 1). Furthermore, there was no significant difference in the expression level of IL-6 receptor among these LNCaP sublines (data not shown).

In the following *in vitro* experiments, almost identical findings were obtained from IL-6-transfected cell lines (LNCaP/IL-6#1 to LNCaP/IL-6#4) or their control cell lines (LNCaP/P and LNCaP/Co); therefore, we subsequently presented the data for LNCaP/IL-6#1 and LNCaP/Co only.

### *In vitro* growth of LNCaP sublines

**Figure 2:**
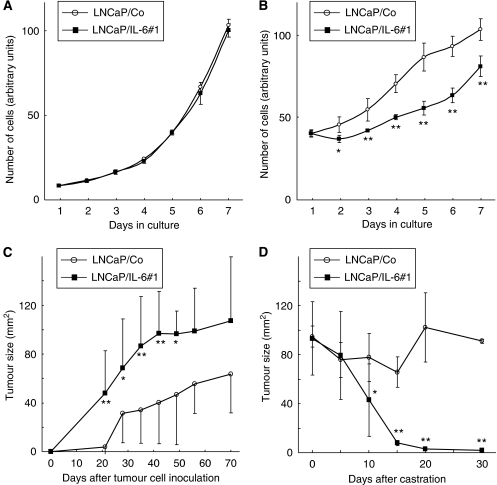
Effect of interleukin-6 (IL-6) overexpression on *in vitro* and *in vivo* cell growth of LNCaP sublines before and after androgen ablation. (**A**) *In vitro* proliferation of LNCaP/Co (control vector-only transfected cell line) and LNCaP/IL-6#1 (IL-6-transfected cell line) cultured in the standard medium was evaluated by counting in triplicate the number of cells in each cell line daily. Bars, s.d. (**B**) Following culture in standard medium for 3 days, the medium was replaced with steroid hormone-depleted charcoal-stripped medium, and *in vitro* proliferation of LNCaP/Co and LNCaP/IL-6#1 under androgen-deprived condition was evaluated by counting in triplicate the number of cells in each cell line daily. Bars, s.d. ^**^ and ^*^, differ from LNCaP/Co (*P*<0.01 and *P*<0.05, respectively). (**C**) Intact nude mice were subcutaneously given 5 × 10^6^ cells of LNCaP sublines in the right flank on day 0. Tumour size was expressed as the product of the maximal diameter multiplied by the perpendicular diameter. Bars, s.d. of tumour size; 10 mice per group. ^**^ and ^*^, differ from LNCaP/Co (*P*<0.01 and *P*<0.05, respectively). (**D**) Following subcutaneous injection of LNCaP sublines, nude mice were castrated on day 0 when the tumour size reached 80 mm^2^ or greater. Tumour size in castrated mice was then serially measured as described above. Bars, s.d. of tumour size; 10 mice per group. ^**^ and ^*^, differ from LNCaP/Co (*P*<0.01 and *P*<0.05, respectively).

To examine the effects of IL-6 overexpression on *in vitro* growth of LNCaP under conditions with and without androgen, the growth of LNCaP sublines cultured in the standard medium and that in CSM were assessed. As shown in Figure 2, there was no significant difference in the growth of LNCaP sublines cultured in the standard medium; however, after androgen deprivation by replacing the standard medium with CSM, the growth of LNCaP/IL-6#1 was significantly inhibited compared with that of LNCaP/Co.

### *In vivo* growth of LNCaP sublines

To examine the *in vivo* effect of IL-6 overexpression on tumour growth, 5 × 10^6^ cells of each cell line were injected subcutaneously into 20 nude mice; then 10 of the 20 mice were castrated when the tumour size reached 80 mm^2^ or greater. In intact mice without castration, the growth of LNCaP/IL-6#1 tumour was significantly faster than that of LNCaP/Co tumour (Figure 2C). However, LNCaP/IL-6#1 tumour in nude mice rapidly regressed after castration, whereas LNCaP/Co tumour growth was transiently inhibited after castration and then continuously accelerated without regulation of androgen (Figure 2D).

### PSA production by LNCaP sublines

**Figure 3:**
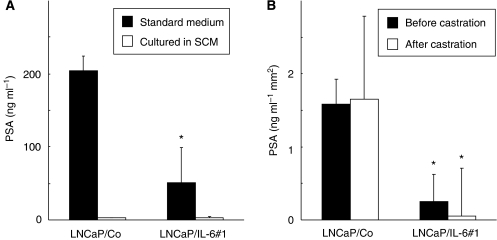
Effect of interleukin-6 (IL-6) overexpression on the production of prostate-specific antigen (PSA) by LNCaP sublines. (**A**) Enzyme-linked immunosorbent assay (ELISA) was performed in triplicate to analyse the concentrations of PSA protein in culture supernatant from LNCaP sublines cultured in both standard medium and steroid hormone-depleted charcoal-stripped medium for 24 h. Bars, s.d. ^*^ differs from LNCaP/Co (*P*<0.01). (**B**) ELISA was performed in triplicate to measure serum PSA levels in nude mice bearing subcutaneous tumours of LNCaP sublines before and 2 months after castration. Bars, s.d. of serum PSA; 10 mice per group. ^*^ differs from LNCaP/Co (*P*<0.01).

As shown in Figure 3A, when maintained in standard medium, PSA concentration in the culture supernatant of LNCaP/IL-6#1 was significantly lower than that of LNCaP/Co; however, under the androgen-deprived condition, PSA concentrations of both LNCaP/Co and LNCaP/IL-6#1 drastically decreased, and there was no significant difference in PSA concentration between these two cell lines. Inconsistent with the *in vitro* finding, serum concentration of PSA, which was adjusted based on tumour volume, in intact mice bearing LNCaP/IL-6#1 tumour before castration was significantly lower than that in mice bearing LNCaP/Co tumour. Two months after castration, PSA concentration in mice bearing LNCaP/IL-6#1 tumour was significantly reduced compared with that before castration, whereas PSA concentration in mice bearing LNCaP/Co tumour acquiring the AI phenotype became greater than that before castration (Figure 3B).

### Effects of exogenous IL-6 treatment on signal transduction pathways in LNCaP sublines

**Figure 4:**
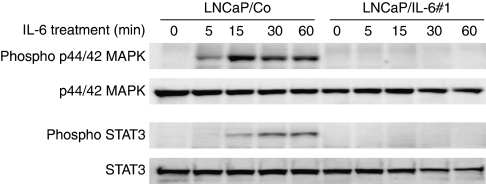
Effects of exogenous interleukin-6 (IL-6) treatment on the signal transduction pathways in LNCaP sublines. LNCaP sublines cultured in serum-free medium was exogenously treated with IL-6 (50 ng ml^−1^), and proteins were extracted from each cell line several times after treatment with IL-6. Both phosphorylated and total p44/42 mitogen-activated protein kinase (MAPK) and signal transducers and activation of transcription 3 (STAT3) protein levels were analysed by western blotting.

To evaluate whether treatment with exogenous IL-6 differentially influences its major downstream signalling pathways, western blot analyses of both phosphorylated and total MAPK, Akt and STAT3 expression in LNCaP sublines were performed. Despite the lack of a significant difference in the expression levels of phosphorylated Akt between LNCaP/Co and LNCaP/IL-6#1 (data not shown), exogenous IL-6 rapidly activated MAPK and STAT3 pathways in LNCaP/Co, but not in LNCaP/IL-6#1; that is, phosphorylated p44/42 MAPK and STAT3, which were not induced in LNCaP/IL-6#1, became detectable in LNCaP/Co 5 and 15 min after treatment with IL-6, respectively (Figure 4).

### Effects of androgen withdrawal on signal transduction pathways in LNCaP sublines

**Figure 5:**
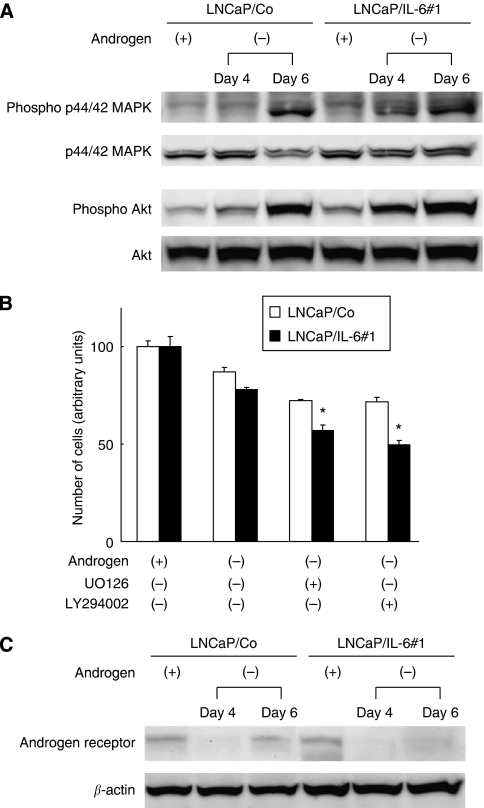
(**A**) Effects of androgen withdrawal on the signal transduction pathways in LNCaP sublines. Proteins were extracted from each cell line cultured in the standard medium for 3 days and that in steroid hormone-depleted charcoal-stripped medium for 4 and 6 days. Both phosphorylated and total p44/42 mitogen-activated protein kinase (MAPK) and Akt protein levels were analysed by western blotting. (**B**) Effect of additional treatment with UO126 or LY294002, a selective inhibitor of mitogen-activated protein kinase kinase or phosphoinositol 3′-kinase on the growth of LNCaP sublines cultured in the androgen-deprived condition. Each cell line cultured in steroid hormone-depleted charcoal-stripped medium for 24 h was treated with UO126 (5 *μ*M) or LY294002 (5 *μ*M). After incubation with either agent for 96 h, cell numbers in each cell line were counted in triplicate. Bars, s.d. ^*^ differs from the growth inhibition by UO126 or LY294002 in LNCaP/Co (*P*<0.01). (**C**) Effects of androgen withdrawal on the expression of androgen receptor (AR) in LNCaP sublines. Proteins were extracted from each cell line cultured in the standard medium for 3 days and that in steroid hormone-depleted charcoal-stripped medium for 4 and 6 days. AR and *β*-actin protein levels were analysed by western blotting.

To determine whether androgen withdrawal modulates the activation patterns of signal transduction pathways through AR, changes in expression of both phosphorylated and total MAPK, Akt and STAT3 in LNCaP sublines were evaluated. There were no significant differences in the expression levels of both phosphorylated and total STAT3 between LNCaP/Co and LNCaP/IL-6#1 under an androgen-deprived condition (data not shown). As shown in Figure 5A, however, androgen withdrawal induced remarkable activation of MAPK and Akt pathways in LNCaP/IL-6#1 compared with that in LNCaP/Co; that is, expression levels of both phosphorylated Akt and p44/42 MAPK in LNCaP/IL-6#1 were significantly upregulated 4 days after androgen withdrawal, whereas there was no upregulation of both phosphorylated Akt and p44/42 MAPK in LNCaP/Co until 6 days after the initiation of culture under an androgen-deprived condition.

Consistent with the evaluation of changes in the downstream signalling pathways of IL-6, we investigated the effect of additional treatment with UO126 or LY294002, a selective inhibitor of mitogen-activated protein kinase kinase (MEK) or PI3K on the growth of LNCaP sublines cultured in the androgen-deprived condition. Treatment with UO126 or LY294002 inhibited the growth of LNCaP/Co and LNCaP/IL-6#1; however, growth inhibitory effects of both UO126 and LY294002 on LNCaP/IL-6#1 were significantly greater than those on LNCaP/Co (Figure 5B).

Changes in the expression of AR in LNCaP sublines were then analysed. As shown in Figure 5C, expression levels of AR in both LNCaP/Co and LNCaP/IL-6#1 were markedly inhibited 4 days after androgen withdrawal; however, AR expression in LNCaP/Co was restored to the basal level 6 days after androgen withdrawal, despite the continuous suppression of AR expression in LNCaP/IL-6 until 6 days after the initiation of culture without androgen.

### Gene microarray analysis in LNCaP sublines

To further address the molecular mechanism involved in the enhanced sensitivity of androgen withdrawal in LNCaP/IL-6#1, the influence of IL-6 overexpression on global gene expression was assessed by gene microarray analysis. We analysed the gene expression profiles in LNCaP/Co and LNCaP/IL-6#1 cultured in both the standard medium and CSM. Excluding genes differentially expressed between LNCaP/Co and LNCaP/IL-6#1 cultured in the standard medium, a total of 223 genes were found to be differentially expressed in LNCaP/IL-6#1 at least 2.0-fold greater or 2.0-fold less than those of LNCaP/Co under an androgen-deprived condition. On the basis of the biological relevance and magnitude of changes, 40 of these 223 genes were selected, and presented in Table 1. For example, MAP3K7 was up-regulated in LNCaP/IL-6#1 compared with that in LNCaP/Co, which is consistent with the findings suggesting the activation of MAPK pathway in LNCaP/IL-6#1, and this might be due to an adaptive change to resist to proapoptotic stimuli induced by androgen ablation. In addition, expression levels of some genes associated with the progression of prostate cancer, such as relaxin, vascular endothelial growth factor (VEGF), vimentin and bone morphogenetic protein-6 (BMP-6) (Soker *et al*, 2001; Dai *et al*, 2005; Thompson *et al*, 2006; Feng *et al*, 2007; Wei *et al*, 2008), were significantly downregulated in LNCaP/IL-6#1 compared with LNCaP/Co. Overall, there are several genes listed in Table 1, which are known to have functions mediating cell growth, apoptotsis, and tumorigenesis, showing expression profiles that are reasonable for theoretically elucidating the molecular mechanism mediating enhanced sensitivity to androgen withdrawal by overexpression of IL-6.

## Discussion

Since reports showing elevated levels of circulating IL-6 in men with advanced prostate cancer (Twillie *et al*, 1995; Drachenberg *et al*, 1999), a number of studies have been performed to clarify how IL-6 signalling is involved in the progression of prostate cancer. Consequently, it has been suggested that crosstalk between AR and IL-6 may have an important function in the activation of AR signalling in a ligand-independent manner (Okamoto *et al*, 1997; Hobisch *et al*, 1998; Lou *et al*, 2000; Lin *et al*, 2001; Ueda *et al*, 2002a, 2002b; Lee *et al*, 2003). To date, however, it remains controversial whether IL-6 confers a malignant phenotype on prostate cancer cells, particularly under an androgen-deprived condition (Okamoto *et al*, 1997; Hobisch *et al*, 1998, 2001; Mori *et al*, 1999; Lou *et al*, 2000; Deeble *et al*, 2001; Lin *et al*, 2001; Ueda *et al*, 2002a, 2002b; Lee *et al*, 2003, 2007; Jia *et al*, 2004). Accordingly, in this study, we established the IL-6-overexpressing LNCaP cells (i.e., LNCaP/IL-6#1), and assessed the effects of IL-6 secreted through an autocrine manner on their phenotype before and after androgen withdrawal.

The growth patterns of LNCaP sublines both *in vitro* and *in vivo* were initially compared. Before androgen ablation, despite the lack of a significant difference in the *in vitro* growth between LNCaP sublines, LNCaP/IL-6#1 tumour grew significantly faster than LNCaP/Co tumour. Under an androgen-deprived condition, however, the *in vitro* growth of LNCaP/IL-6#1 was significantly suppressed compared with that of LNCaP/Co. This enhanced sensitivity of LNCaP/IL-6#1 to androgen withdrawal was more remarkable *in vivo*; that is, after castration, LNCaP/IL-6#1 tumour rapidly regressed and completely disappeared. According to previously reported studies, the effects of IL-6 on the growth of LNCaP cells seem to be puzzling with some groups showing growth stimulation, whereas others showing growth inhibition (Spiotto and Chung, 2000; Lee *et al*, 2003, 2007; Jia *et al*, 2004). For example, Lee *et al* (2007) reported the bifunctional action of IL-6 according to the manner of its secretion; that is, IL-6 acts as a growth inhibitor for LNCaP cells by a paracrine mechanism, whereas endogenously produced IL-6 stimulates LNCaP cell growth by an autocrine mechanism. As most of the previously observed effects of IL-6 on the growth of prostate cancer cells were examined *in vitro*, this study is the first to demonstrate the following evidence that overexpression of IL-6 in LNCaP cells enhanced their sensitivity to androgen withdrawal both *in vitro* and *in vivo*, resulting in marked growth inhibition immediately after androgen ablation. Furthermore, it would be of interest to investigate the effects of an antiandrogen, such as bicalutamide, on the growth of LNCaP sublines.

PSA is a reliable tumour marker showing a proportional change in relation to the degree of prostate cancer progression (Lilja *et al*, 2008). In this study, PSA concentrations in culture supernatants and sera from mice generally reflected the proliferative status of LNCaP sublines cultured *in vitro* and that subcutaneously implanted, respectively, under both conditions with and without androgen. Interestingly, the amount of baseline PSA production by LNCaP/IL-6#1 was significantly smaller than that by LNCaP/Co both *in vitro* and *in vivo*, when androgen was supplemented; however, several earlier studies have shown that treatment of LNCaP cells with IL-6 enhances their AR-mediated PSA gene expression through an increase in PSA reporter activity (Lin *et al*, 2001; Ueda *et al*, 2002a; Lee *et al*, 2003). Although the discrepancy described above cannot be clearly explained, it would be speculated that overexpression of IL-6 by LNCaP cells itself rather than exogenous administration of IL-6 may render them undifferentiated, resulting in a lesser amount of PSA production than that in LNCaP/Co cells.

Considering the unique proliferative potential and PSA production of LNCaP/IL-6#1 cells, it would be of interest to investigate whether LNCaP/IL-6#1 exhibits a different response to exogenous IL-6 compared with that of LNCaP/Co. As reported earlier (Ueda *et al*, 2002a; Jia *et al*, 2004), exogenous IL-6 induced rapid activation of MAPK and STAT3 pathways in LNCaP/Co cells, which are unable to secrete endogenous IL-6. In addition, rapid activation of MAPK pathway by IL-6 was also observed in a subline of LNCaP derived by chronic treatment with IL-6 (Steiner *et al*, 2003). However, treatment of LNCaP/IL-6#1 with exogenous IL-6, which was a markedly greater amount than that produced by LNCaP/IL-6#1, failed to activate the MAPK and STAT3 pathways. Collectively, these findings strongly suggest that IL-6 may exert various effects on changes in the phenotype of prostate cancer cells through different molecular mechanisms according to the manner of secretion.

Another point of interest is the mechanism whereby overexpression of IL-6 enhances sensitivity of LNCaP cells to androgen withdrawal. To address this point, it was investigated whether the downstream signalling pathways of IL-6 are differentially regulated by androgen between LNCaP sublines. Although the growth of LNCaP/IL-6#1 was significantly inhibited compared with that of LNCaP/Co under an androgen-deprived condition, Akt and MAPK pathways in LNCaP/IL-6#1, which are generally regarded as a positive regulator of prostate cancer cell growth through the transactivation of AR (Ueda *et al*, 2002b; Corcoran and Costello, 2003; Lee *et al*, 2003), appeared to be markedly activated following androgen withdrawal compared with those in LNCaP/Co. However, stress-induced increase in several antiapoptotic genes, such as bcl-2, clusterin, insulin-like growth factor binding protein-2 (IGFBP-2), IGFBP-5 and heat-shock protein 27, have been shown to have important functions in the AI progression of prostate cancer after castration (Gleave *et al*, 1999; Miyake *et al*, 2000b, 2000c; Kiyama *et al*, 2003; Rocchi *et al*, 2004). Therefore, these findings suggest that activation of Akt and MAPK pathways after a proapoptotic trigger in LNCaP/IL-6#1 represents an adaptive mechanism mediating cell survival. In addition, this hypothesis was supported by the finding that additional treatment with UO126 or LY294002, a selective inhibitor of MEK or PI3K, respectively, after androgen deprivation resulted in significant growth inhibition of LNCaP/IL-6#1 compared with that of LNCaP/Co. Furthermore, the restoration of AR expression after androgen withdrawal in LNCaP/IL-6#1 was shown to be significantly delayed compared with that in LNCaP/Co; however, the unique proliferative potential and PSA production in LNCaP/IL-6#1 could not be entirely explained by the changes in AR expression in this cell line.

We further investigated the molecular mechanism underlying the enhancement of androgen withdrawal-induced apoptotic cell death of LNCaP/IL-6#1 using gene microarray analysis. Gene expression profiling showed that 223 genes were differentially expressed between LNCaP/Co and LNCaP/IL-6#1 under androgen-deprived condition, and 40 of these 223 were listed considering the biological relevance and magnitude of changes. These outcomes were not confirmed by an alternative method; however, expression profiles in some genes in LNCaP/Co before and after androgen withdrawal were consistent with those in parental LNCaP reported in earlier studies (Stewart *et al*, 2001; Thompson *et al*, 2006), suggesting the reliability of our gene microarray analysis. Although it would be necessary to perform functional analysis to identify genes having potential functions in the mechanism described above, several candidate genes could be identified by gene microarray analysis. For example, androgen withdrawal-induced marked downregulation of relaxin, VEGF, vimentin and BMP-6, involved in cancer progression (Soker *et al*, 2001; Dai *et al*, 2005; Thompson *et al*, 2006; Feng *et al*, 2007; Wei *et al*, 2008) as well as upregulation of cellular retinol binding protein 7 and interferon-*γ* receptor, negatively regulating cancer progression (Kuppumbatti *et al*, 2001; Yang *et al*, 2008), in LNCaP/IL-6#1. In addition, DnaJ, acting as a cytoprotective chaperon in response to stress-induced apoptosis (Cajo *et al*, 2006), were upregulated in LNCaP/IL-6#1, which might be an adaptive change as observed in Akt and MAPK pathways after androgen deprivation.

In conclusion, this is the first study to show enhanced sensitivity to androgen withdrawal due to overexpression of IL-6 in AD human prostate cancer LNCaP cells. Furthermore, the present findings suggest that the difference in response to exogenous IL-6 between LNCaP/Co and LNCaP/IL-6#1 may, at least in part, be involved in the differential sensitivity to androgen withdrawal between these two sublines, and that despite the significant activation of Akt and MAPK pathways in LNCaP/IL-6#1 compared with that in LNCaP/Co under an androgen-deprived condition, this might be due to the response to proapototic stimuli representing an adaptive cell survival mechanism. Although gene microarray analysis identified several potentially important candidate genes responsible for the different sensitivities to androgen ablation between LNCaP/Co and LNCaP/IL-6#1 by globally evaluating their gene expression profiles, it would be further required to accumulate the intensive investigation to elucidate the molecular mechanism underlying changes in the phenotype of LNCaP cells by overexpression of IL-6.

## Figures and Tables

**Table 1:** Gene microarray analysis in LNCAP sublines under androgen-deprived condition

**Gene symbol**	**Genebank accession no.**	**Gene name**	**Fold difference^a^**
KCNJ3	BC022495	Potassium inwardly rectifying channel, subfamily J, member 3	23.40
CBLN2	BC035789	Cerebellin 2 precursor	21.69
DNAJC15	BC010910	DnaJ (Hsp40) homolog, subfamily C, member 15	16.99
KHDC1	BC022080	KH homology domain containing 1	9.21
GRIK2	BC063814	Glutamate receptor, ionotropic, kainate 2	8.97
CDH18	BC031051	Cadherin 18, type 2	8.28
GABRG2	BC069348	Gamma-aminobutyric acid (GABA) A receptor, gamma 2	8.16
AMOTL1	BC037539	Angiomotin like 1	3.79
IFNGR2	BC003624	Interferon gamma receptor 2 (interferon gamma transducer 1)	3.09
HOXC12	AH010514	Homeobox C12	2.95
ARHGEF11	BC057394	Rho guanine nucleotide exchange factor (GEF) 11	2.73
USP51	BC035907	Ubiquitin specific peptidase 51	2.63
ABCC4	BC041560	ATP-binding cassette, sub-family C (CFTR/MRP), member 4	2.51
SATB2	BC098136	SATB homeobox 2	2.48
NAMPT	BC072439	Nicotinamide phosphoribosyltransferase	2.42
DYNC1I1	BC064521	Dynein, cytoplasmic 1, intermediate chain 1	2.34
FBLN7	BC035784	Fibulin 7	2.25
RBP7	BC063013	Retinol binding protein 7, cellular	2.21
C1QL1	BC008798	Complement component 1, q subcomponent-like 1	2.13
MAP3K7	BC017715	Mitogen-activated protein kinase kinase kinase 7	2.05
RASAL1	BC093724	RAS protein activator like 1 (GAP1 like)	−2.10
VEGFA	BC065522	Vascular endothelial growth factor A	−2.16
HIST1H2BO	BC106720	Histone cluster 1, H2bo	−2.29
HIST1H2BH	BC096116	Histone cluster 1, H2bh	−2.31
GPRC5C	BC110848	G protein-coupled receptor, family C, group 5, member C	−2.33
BMP6	BC112352	Bone morphogenetic protein 6	−3.01
SH3GL3	BC042864	SH3-domain GRB2-like 3	−3.07
RGS16	AF009356	Regulator of G-protein signaling 16	−3.99
HRK	NM003806	Harakiri, BCL2 interacting protein (contains only BH3 domain)	−4.05
TCF4	BC125084	Transcription factor 4	−4.40
EPS8	BC030010	Epidermal growth factor receptor pathway substrate 8	−5.32
PPP1R1A	BC022470	Protein phosphatase 1, regulatory (inhibitor) subunit 1A	−8.08
HOXA13	BC075791	Homeobox A13	−8.28
MAF	BC081542	v-maf musculoaponeurotic fibrosarcoma oncogene homolog (avian)	−9.39
PTPRB	BC051329	Protein tyrosine phosphatase, receptor type, B	−9.55
RLN1	BC005956	Relaxin 1	−11.33
VIM	M14144	Vimentin	−18.81
ACSS1	BC039261	Acyl-CoA synthetase short-chain family member 1	−28.42
S100A10	BC105786	S100 calcium binding protein A10	−37.45
RGS2	BC042755	Regulator of G-protein signaling 2, 24 kDa	−89.77

a The average magnitude of difference in each gene expression in LNCaP/IL-6#1 relative to that in LNCaP/Co.
